# COVID-19 Vaccine Hesitancy among the Public in Kuwait: A Cross-Sectional Survey

**DOI:** 10.3390/ijerph18168836

**Published:** 2021-08-22

**Authors:** Jumana Alibrahim, Abdelmoneim Awad

**Affiliations:** Department of Pharmacy Practice, Faculty of Pharmacy, Kuwait University, Safat, P.O. Box 24923, Kuwait City 13110, Kuwait; jomana.alibrahim@hscp.ku.edu.kw

**Keywords:** coronavirus disease, COVID-19, vaccine, COVID-19 vaccine, vaccine hesitancy, general population, Kuwait

## Abstract

Vaccine hesitancy (uncertainty or unwillingness to receive vaccinations) is a major barrier to manage the COVID-19 pandemic in the long term. This study aimed to explore the prevalence of COVID-19 vaccine hesitancy among the public in Kuwait, to understand their attitudes towards vaccines in general, and to identify predictors of COVID-19 vaccine hesitancy. A cross-sectional study was conducted among 4147 adults aged ≥ 18 years. The snowball sampling strategy was used for data collection through social media and e-mails. A total of 3061 (73.8%) respondents indicated that they were vaccinated or intending to be vaccinated against the COVID-19 infection, while 1086 (26.2%) expressed their vaccine hesitancy. The most common reasons for vaccine hesitancy were the concerns about the vaccine’s possible side effects, its rushed development, and its efficacy in preventing the infection. Over half (57.2%) of respondents expressed intermediate to high levels of negative attitude towards vaccines in general. On the multivariable logistic regression analysis, the findings revealed that vaccine hesitancy was significantly more prevalent among respondents aged 30–64 years; females; married or divorced; residents of Hawalli, Al-Farwaniyah, Al-Jahra, and Mubarak Al-Kabeer; had intermediate monthly average income; non-smokers; not feeling worried about catching the infection; do not know whether any of their family members have been infected or died because of COVID-19 infection; do not have a large extent of confidence in the Kuwait health system’s ability to handle the pandemic; did not receive influenza vaccine during the last year; refused or elected to forego a doctor-recommended vaccine; did not receive adequate information from the public health authorities/healthcare providers about the COVID-19 vaccines; none of their first-degree family members received or were intending to receive the vaccine; and expressed intermediate to high levels of negative attitudes towards vaccines in general. The present findings provide a steer as to the groups that most need to be reached to increase the rates of vaccine uptake.

## 1. Introduction

The Coronavirus Disease 2019 (COVID-19) is an infectious disease that is caused by a new SARS-CoV-2 (severe acute respiratory syndrome coronavirus 2) [1]. It appears to cause a wide range of symptoms, including asymptomatic infections, mild infections of the upper respiratory tract, severe viral pneumonia, respiratory failure, multiple organ failure, and death [2]. Due to the alarming levels of spread worldwide and the severity of the infection, the World Health Organization (WHO) officially announced the COVID-19 as a pandemic in March 2020 [3]. This had led to the global implementation of various control measures to prevent the spreading of the virus, such as limiting social gatherings, social distancing, wearing face masks, and partial and comprehensive lockdowns. As of 25 July 2021, there were 194,567,100 confirmed cases of COVID-19 and 4,171,596 deaths worldwide, with about 391,781 cases and 2,279 deaths in Kuwait [4].

Reducing the COVID-19 related morbidity and mortality will depend on the availability of safe and effective COVID-19 vaccines. Additionally, vaccines must be available globally and widely accepted and received by the public to assure their immunological protection [5]. The WHO issued an Emergency Use Listing for Pfizer-BioNTech COVID-19 vaccine on 31 December 2020; two AstraZeneca/Oxford COVID-19 vaccines on 15 February 2021, produced by AstraZeneca-SKBio (Korea) and the Serum Institute of India; COVID-19 vaccine Ad26.COV2.S was developed by Janssen (Johnson & Johnson) on 12 March 2021; and Moderna vaccine on 31 April 2021; and the Sinopharm COVID-19 vaccine was developed by Sinovac/China National Pharmaceutical Group on 7 May 2021 [6].

As of 24 July 2021, a total of 3,646,968,156 doses of vaccines have been administered worldwide according to the WHO [7]. The two currently available COVID-19 vaccines in Kuwait are Pfizer-BioNTech and AstraZeneca-Oxford. The Kuwait Ministry of Health recently announced that since the campaign started on 27 December 2020, as of 3 July 2021, 1 million and 452,148 individuals received one dose and 923,307 individuals received two doses. This indicates that by September 2021, about 50% of the total population would have completed their vaccination. A significant acceleration of the vaccination rate is expected with the arrival of the Moderna and Johnson & Johnson vaccines in the last quarter of this year. This will allow the vaccination of 3 million people (70% of the population) to be attained by the end of this year [8]. Despite the enormous positive impact of immunization against infectious diseases and improving health outcomes, vaccine refusal among the general public persists, which presents a major threat to a healthy community as it undermines society’s protection against vaccine-preventable infections [9]. Vaccine hesitancy, which refers to people who may be uncertain or unwilling to receive a vaccine, presents a major challenge to the success of vaccination programs [10]. The impact of vaccine hesitancy on parental acceptance of routine childhood vaccines has been recognized globally as a growing problem. Previous studies assessed vaccine hesitancy and its associated factors among parents towards childhood vaccination and reported that it ranged from 7.7% to 34.7% [11,12,13]. Vaccine hesitancy was found to be significantly more common among those who were concerned about vaccine safety, received information from the mass media, did not receive adequate knowledge from pediatricians on childhood vaccinations, not perceiving their infants to be at high risk for the vaccine-preventable diseases, and agreed with political leaders who oppose vaccination, agreed that childhood vaccinations are primarily a profitable business for pharmaceutical companies [11,12,13]. In 2019, the WHO listed vaccine hesitancy as one of the ten threats to global health [14]. Hence, it is crucial to understand the reasons and predictors for vaccine hesitancy to guide public health vaccination strategies.

Several surveys regarding the intention to receive the COVID-19 vaccine among the general public worldwide have been published [15,16,17,18,19,20,21,22,23,24,25,26,27,28,29,30,31,32,33,34,35,36,37,38,39,40]. These studies have explored the prevalence of COVID-19 vaccine acceptance when it will be available, and the determinants for intention to accept the vaccine. The rates of COVID-19 vaccine acceptance in these studies ranged between 23.6% and 97%. One study has reported the general population’s willingness to receive the COVID 19 vaccine in the United States of America (USA) before and after its availability; it revealed a significant decline in vaccine hesitancy among the public by 10.8% from 46% in October 2020 to 35.2% in March 2021 [41]. Since vaccine acceptancy is context-specific and varies with geography, culture, and sociodemographic characteristics, further studies are needed to address the scope of the COVID-19 vaccine hesitancy, particularly in the Middle East and North Africa, Sub-Saharan Africa, Eastern Europe, Central Asia, and the Middle and South America [15]. Limited studies were reported from the Middle Eastern region [18,19,20,21,22,23]. Three manuscripts were published from studies conducted in Kuwait among the general public before the availability of COVID-19 vaccines. The first study was performed during the period from May to August 2020 among 7241 adults aged ≥ 21 years; 67% of them agreed to be vaccinated during the entire lockdown period. However, it significantly decreased over time to about 50%, when the COVID-19 related restrictions were lessened [18]. The second study was conducted during the period from August to September 2020 among adults aged ≥18 years; 53.1% of them indicated their willingness to be vaccinated [19]. The last study was performed in December 2020 among 771 adults aged ≥16 years; 23.6% of them indicated their intention to be vaccinated against the COVID-19 infection [20]. Hence, this study is designed to build upon previous research by providing more recent data after the availability of the COVID-19 vaccines in Kuwait. It aimed to explore the prevalence of the COVID-19 vaccine hesitancy among the public in Kuwait, understand the public’s general attitudes towards a vaccine in general, and identify the predictors of vaccine hesitancy (i.e., uncertainty or unwillingness to receive a COVID-19 vaccine). The findings of the present study may help develop strategies for improving the vaccination program in Kuwait.

## 2. Materials and Methods

### 2.1. Study Area and Design

Kuwait is a Middle Eastern country with an area of 17,820 km^2^ and an estimated total population of 4,332,621 people (2021 estimate) [42]. A descriptive and cross-sectional survey-based study was conducted among the general population in Kuwait who are aged ≥18 years. It was performed during the period from 26 March to 26 April 2021. In Kuwait, the administered vaccine doses reached a total of 576,204 on 26 March and 1,040,777 on 26 April 2021 [43] This study was approved by the Ministry of Health Research Ethical Committee, Kuwait (MoH/REC/1344).

### 2.2. Study Population

The sample size was determined using PS: Power and Sample Size Calculator V.3.1.6 [44]. A minimum sample of 1120 individuals would be necessary to determine a 10% difference in proportion between two groups, for example, male versus female with 90% power and 5% significance level. The inclusion criteria were an age of ≥18 years and being a resident in Kuwait. A type of convenience sample, which is the snowball sampling strategy, was used for data collection. The invitation and the survey link were shared through social media (WhatsApp, Twitter, Instagram, Telegram) and emails. The survey objectives, maintenance of confidentiality, anonymity, and voluntary participation were elucidated in the invitation. On receiving and clicking the link, the invited candidates were auto-directed to the informed consent page, followed by the survey questions. The participants should indicate at the end of the consent page either agreeing or disagreeing to participate in the study. The invited participants were requested to roll out the questionnaire further to their social media and email contacts.

### 2.3. Study Questionnaire

The study questionnaire was mainly adapted from a validated survey that was previously used among the general population in the United Kingdom (UK) to assess attitudes towards vaccines and intention to vaccinate against COVID-19 [32]. The adapted survey was tailored to suit the local population and assure its applicability in Kuwait. The face and content validity of the adapted survey was established by three researchers from the Department of Pharmacy Practice at Kuwait University. The questionnaire was translated into Arabic, and its accuracy and meaning with the English version were checked by four academic staff members at Kuwait University who are fluent in both English and Arabic languages. Minor recommended modifications were included where necessary. Then it was pretested among 10 individuals, who were not included in the study sample, for content, readability, and understanding, and minor amendments were included. Incentives were not offered for the completion of the questionnaire.

The survey consisted of 41 items. The first 12 items were used to collect information about sociodemographic characteristics (age, gender, marital status, educational level, employment, residence, area, income, and working or studying in the medical field) and other items related to overall health, smoking, and chronic diseases. The following 9 items were included to collect information about the COVID-19 infection/pandemic regarding feeling worried about catching the infection, being infected themselves or any of their family members with COVID-19, any of their family members have died due to COVID-19 infection, sources of information about COVID-19, knowledge level on COVID-19 infection, the extent of following recommendations to prevent the spread of COVID-19, and extent of confidence in Kuwait’s Government and Kuwait’s health system to handle the COVID-19 pandemic well. The following 2 items were used to collect information about vaccination behavior (had taken an influenza vaccine in the prior year and had ever refused or elected to forego a doctor-recommended vaccine for themselves or someone they are responsible for). The following 6 items included information related to COVID-19 vaccines (awareness about the availability of multiple vaccines, received adequate information from the health authorities/health care provider about vaccines available in Kuwait, first-degree family members received or intending to receive the vaccine, had received the vaccine, intention to receive the vaccine if had not been vaccinated, and perceived reasons if unsure or unwilling to take the vaccine). The last part of the survey contained the 12-item Vaccination Attitudes Examination (VAX) scale [45]. The study population was asked to focus on vaccines in general, not specifically on the COVID-19 vaccine. Participants indicated their responses on a six-point scale from 1 “strongly agree” to 6 “strongly disagree” [45].

### 2.4. Statistical Analysis

The Statistical Package for Social Sciences (IBM SPSS Statistics for Windows, version 27) was used for data entry and analysis. The findings were presented as percentages (95% confidence intervals; CI), medians (interquartile range-IQR), and means (standard deviation-SD). The continuous data of the age and VAX scale responses were found to be not normally distributed by using the Shapiro–Wilk and Kolmogorov–Smirnov tests. The described four subscales of the VAX scale were calculated and reported as follows: (1) mistrust of vaccine benefit (items 1 to 3), (2) worries about unforeseen future effects (items 4 to 6), (3) concerns about commercial profiteering (items 7 to 9), and (4) preference for natural immunity (items 10 to 12). The Cronbach’s alphas for the internal reliability were reported as 0.77–0.93 for the four subscales in previous studies [45,46]. The levels of negative attitudes towards vaccines based on the four subscales and the overall VAX scale were classified into high (score 5–6), intermediate (score 3–4), and low (score 1–2) [32]. Based on this classification, items 4 to 12 were reverse coded for the calculation of the scores.

The purposeful selection strategy of variables was used for the model building. The first step was to explore the unadjusted association between the respondent’s sociodemographic characteristics, their responses to the items related to COVID-19 infection and vaccines, and their attitudes towards vaccines with the outcome variable (vaccine hesitancy) using the univariate logistic regression. All variables with *p* < 0.25 in the univariate analysis were included in the multivariable logistic regression analysis to identify the independent variables, which are good predictors for vaccine hesitancy (uncertainty or unwillingness to vaccinate against COVID-19). A cutoff value of 0.25 is supported by the literature [47,48]. The second step was to fit the multivariable model comprising all variables identified in step one. Education, perceived overall health, and obtaining information about COVID-19 infection from the scientific websites and articles were excluded and a new model was fitted, because these did not contribute to the model with *p*-values 0.763, 0.509, and 0.671, respectively. These two models were compared by using the partial likelihood ratio test to ensure that the parsimonious model fits as well as the original model. In the parsimonious model, the coefficients of variables were compared to coefficients in the original one. The change of coefficients (Δβ) was <15%, which indicated that the excluded variables were not important in the sense of providing a needed adjustment for one or more of the variables remaining in the model. The potential interactions between the covariates were checked in the third step. The results revealed that the *p*-values for interaction were far from the significance level. The final step was the use of the Hosmer–Lemeshow goodness of fit test for assessing the fit of the model. The results revealed chi-squared = 4.901, df = 8, *p*-value = 0.768. A p-value of 0.768 indicated that there was no significant difference between observed and predicted values; hence, a good logistic regression model fit. The results of the multivariable logistic analysis are reported with the odds ratio (OR) and 95%CI. A probability level of *p* < 0.05 based on two-sided tests was considered statistically significant. The dependent variable was coded as (received the vaccine or intending to receive it = 0) and (uncertain or unwilling to receive the vaccine = 1). The independent variables were coded as presented in the table showing the results of the multivariable logistic regression analysis in the results section.

## 3. Results

### 3.1. Sociodemographic and Other Characteristics of Respondents

A total of 4147 adults participated in this study. Their median (IQR) age was 35 (25) years [mean (SD) 36.5 (14); range 18–80]. Of the 4147 participants, 75.4% were females, 55.6% were married, and 77.7% had university and postgraduate degrees. More than half (53.6%) were employed, 57.9% were from the Capital and Hawalli, 53.3% had a monthly average income >1000 KD, and 24.6% work or study in the medical field. Most of the participants (93.3%) rated themselves to have very good and good overall health, 86.4% were non-smokers, and 78.4% had no chronic diseases. Table 1 shows the sociodemographic and other characteristics of the respondents.

### 3.2. Information Related to the COVID-19 Infection

More than half of the participants (59.3%) felt worried about catching the COVID-19 infection, 82.2% have not been or do not know if they were infected with COVID-19, and 66.4% have family members who have been infected with COVID-19. A family member who died because of a COVID-19 infection was reported by 19.5% of participants. The common three sources that were reported to be utilized to obtain information about COVID-19 infection were social media (68.7%), mass media (48.9%), and healthcare providers (45.2%). More than two-thirds (71.5%) of the participants rated their knowledge level as very good and good on the COVID-19 infection, and 53.2% reported following the recommendations from the health authorities to a large extent to prevent the spread of COVID-19 infection. A moderate extent of confidence in Kuwait’s government and Kuwait’s health system’s ability to handle the COVID-19 pandemic was indicated by 39.2% and 41.9%, respectively. Table 2 shows the results about the answers to questions related to COVID-19 infection.

### 3.3. Information Related to Vaccines

One thousand, two hundred and eighteen (29.4%) respondents indicated that they received the influenza (flu) vaccine during the last 12 months. The majority of the participants (*n* = 3879, 93.5%) never refused or elected to forego a doctor-recommended vaccine for themselves or someone they were responsible for, such as their children. Most of the respondents (*n* = 4018, 96.9%) were aware that multiple vaccines were available for COVID-19. About two-thirds (*n* = 2657, 64.1%) reported that they received adequate information from the public health authorities or their healthcare provider about the COVID-19 vaccines available in Kuwait. In addition, 91% (*n* = 3775) reported that one of their first-degree family members received or intended to receive the COVID-19 vaccine.

### 3.4. Acceptance of the COVID-19 Vaccine

Two thousand four hundred eighty-two (59.8%; 95% CI: 58.3–61.3) participants reported that they had received the COVID-19 vaccine, 579 (14.0.0%; 95% CI: 12.9–15.1) indicated their intention to vaccinate, 607 (14.6%; 95% CI: 13.6–15.8) were uncertain whether to vaccinate or not, and 479 (11.6%; 95% CI: 10.6–12.6) indicated their unwillingness to vaccinate. Hence, a total of 3061 respondents (73.8%; 95% CI: 72.4-75.1) indicated that they were vaccinated or intended to vaccinate, while 1086 (26.2%; 95% CI: 24.9–27.6) expressed vaccine hesitancy (i.e., uncertainty or unwillingness to receive the vaccine). Table 3 shows the reasons for uncertainty or unwillingness to vaccinate. The three most common reasons are the concerns about the possible side effects of the COVID-19 vaccine (75.6%), the development of the COVID-19 vaccine was rushed and has not been thoroughly tested before approval (53%), and concerns about the efficacy of the COVID-19 vaccine in prevention from getting the infection (48.4%). Table 3 presents the reasons for uncertainty or unwillingness to vaccinate.

### 3.5. Attitudes towards Vaccination

Table 4 presents the responses to the 12 items of the VAX scale. Respondents expressed a low level of negative attitude (median score 1 to 2) towards the following five items: vaccination programs are a big con, feeling safe after being vaccinated, can rely on vaccines to stop serious infectious diseases, feel protected after getting vaccinated, and authorities promote vaccination for financial gain, not for people’s health. An intermediate level of negative attitude (median score 3 to 4) was expressed by the participants towards the remaining seven items.

Table 5 shows the four VAX subscales. The Cronbach’s alphas for the mistrust of vaccine benefit, worries about unforeseen future effects, concerns about commercial profiteering, and preference for natural immunity were 0.93, 0.79, 0.79, and 0.81, respectively; and for overall VAX score it was 0.89. The participants expressed a low level of negative attitude (median score 2) towards the mistrust of vaccine benefits and concerns about commercial profiteering. An intermediate level of negative attitude (median score 3 to 4) was expressed by the participants towards the preference for natural immunity and worries over unforeseen future effects. The overall median (IQR) VAX score was 2 (3), reflecting an overall low level of negative attitude towards vaccines among the study population.

### 3.6. Predictors of Uncertainty and Unwillingness to Vaccinate against the COVID-19 Infection

The multivariable logistic regression analysis revealed that 18 out of 31 variables were significantly and independently associated with the respondent’s uncertainty or unwillingness to vaccinate against the COVID-19 infection. It was found that uncertainty or unwillingness to vaccinate was significantly higher (*p* < 0.05) among respondents aged 30-64 years; females, those who are married or divorced; residents of Hawalli, Al-Farwaniyah, Al-Jahra, and Mubarak Al-Kabeer; those who had intermediate monthly average income (500–1000 KD); non-smokers; those who are not feeling worried about catching a COVID-19 infection; those who do not know whether any of their family members have been infected with COVID-19 or died because of COVID-19 infection; those who do not have a large extent of confidence in Kuwait health system’s ability to handle the COVID-19 pandemic well; those who have not received influenza vaccine during the last year; those who refused or elected to forego a doctor-recommended vaccine for them or someone they are responsible for; those who did not receive adequate information from the public health authorities/their healthcare providers about the COVID-19 vaccines available in Kuwait; those who none of their first-degree family members received or are intending to receive the COVID-19 vaccine; and those who expressed high and intermediate levels of negative attitudes towards mistrust of vaccine benefit, concerns about commercial profiteering, preference for natural immunity, and the overall VAX score. Table 6 presents the multivariable logistic regression for predicting uncertainty and unwillingness to vaccinate against the COVID-19 infection.

## 4. Discussion

This study appears to be the first to estimate the prevalence of the COVID-19 vaccine acceptance among the general public in Kuwait after the vaccine has already been available and the vaccination program started in December 2021, and likely in the Middle Eastern region. Additionally, it is the fourth study to be conducted in Kuwait to assess the acceptance of the COVID-19 vaccine among the general public. The previous three studies were performed during the period from May to December 2020 to assess the potential acceptance when the COVID-19 vaccine will be available in Kuwait [18,19,20]. The present findings provide more recent data that reflect the difference in the COVID-19 vaccine acceptability among the general population after it has been available in Kuwait and a better understanding of the reasons and predictors for the COVID-19 vaccine hesitancy (uncertainty or unwillingness to vaccinate). These results are necessary and may help the health authorities in the design of appropriate strategies to enhance the general public’s willingness to vaccinate in Kuwait. Moreover, the present results allow for essential comparison with current and future research work in the Middle Eastern countries, and worldwide.

Almost three-quarters (73.8%) of the participants reported the acceptance (vaccinated or intending to vaccinate) of the COVID-19 vaccine, which is close to previous findings in France, Italy), the UK, Australia, and the USA, which ranged from 69% to 80% [26,27,31,33,38]. Although the present finding is considered to be high, it was lower than in Indonesia (93.3%), Ecuador (97.0%), Malaysia (94.3%), and China (91.3%) [15,25]. However, it was higher than in Jordan, Saudi Arabia, Turkey, China, Germany, Italy, France, Greece, Poland, Ireland, the UK, Russia, and the USA, which ranged between 28.4% and 66% [15,20,21,22,23,24,29,30,31,32,34,35,36,37]. In comparison with previous studies in Kuwait, the acceptance of the COVID-19 vaccine in this study was higher, as only 67% of the participants in the study conducted from May to August 2020 agreed to get vaccinated during the complete lockdown, then it decreased to 50% after the restrictions were lessened [18]. Additionally, only 53.1% and 23.6% were willing to vaccinate in the other previous two studies conducted from August to September 2020 and in December 2020, respectively [19,20]. The variability of the threshold for COVID-19 herd immunity among countries was previously estimated with a proposed average threshold of around 67.0% [49]. Hence, the present finding that 73.8% of participants were vaccinated or intending to vaccinate is considered a high level of acceptability compared to previous studies in Kuwait, which may highlight that Kuwait may reach the threshold for herd immunity against COVID-19 by the end of this year, as a recent report indicated that by the end of September about 50% of the total population would have been vaccinated [8]. This high level of acceptability may be due to the overall low to intermediate levels of negative attitude towards vaccines in general, which was reported by 75.5% of respondents. Other possible explanations may be that about three-fifths of participants (59.3%) feel worried about catching the COVID-19 infection and 71.5% rated their knowledge level as very good and good on the COVID-19 infection.

On the other hand, 26.2% of participants reported their hesitancy toward the COVID-19 vaccine, which is close to previous studies performed in France (28.8%), Northern Italy (31.1%), and the UK (31.0%) [26,28,31]. It is lower than that in Jordan, Saudi Arabia, China, Germany, Greece, Ireland, the UK, and the USA, which ranged from 35.2 and 71% [21,22,23,24,29,30,31,32,34,41], but higher than in Australia (19.8%) [33]. It is worth stating that the present COVID-19 vaccine hesitancy (26.2%) is lower than that reported in the three previous studies among the general population in Kuwait after the complete lockdown, which ranged from 47% to 76.4% [18,19,20]. The reasons reported by almost one-third or more of those who are uncertain or unwilling to vaccinate were the concerns about the possible side effects of the COVID-19 vaccine, the development of the vaccine was rushed and has not been thoroughly tested, concerns about its efficacy in prevention from getting the infection, the vaccine has not yet been taken by many people to know its side effects, inadequate information about the vaccine, and some of the physicians posted videos on social media advising against receiving the vaccine which raised their suspicion. These barriers were also reported by previous similar studies [19,21,24,32]. These findings highlight the need for the health authorities to design effective educational interventions for addressing these concerns at the general public level in Kuwait.

Over half of the respondents (57.2%) expressed an intermediate to a high level of negative attitude towards vaccines in general. Comparing the present results with a study conducted in the UK using the VAX scale revealed that the percentages of respondents who expressed a low level of negative attitude towards the four subscales were lower than in the UK study: mistrust of vaccine benefit (55.8% vs. 75.6%), worries over unforeseen future effects (22.1% vs. 30.8%), concerns about commercial profiteering (55.2% vs. 63.1%), and preference for natural immunity (38.2% vs. 46.7%) [32]. The current results highlight the need for improving the public attitudes towards vaccines in general through effective educational interventions.

The present study identified several predictors for hesitancy to vaccinate against the COVID-19 infection. Vaccine hesitancy was found to be more prevalent among respondents aged 30–49 years and 50–64 years, which is in agreement with a previous study in the UK [32] and close to previous studies in Kuwait ≥ 30 to 74 years [18] and 55–64 years [19]. It is worth noting that the younger study participants (18–29 years) did not show significantly higher vaccine hesitancy compared to the older respondents aged ≥65 years, which is consistent with prior studies in Kuwait [19] and the USA [39]. It is evident that the elderly individuals accepted the vaccination more than the younger due to the reports that the COVID-19 infection has a more severe effect on the elderly [28], hence, further qualitative research is required to provide a better understanding of the reasons behind the low vaccine hesitancy among those aged 18-29 years in Kuwait. Females were found to be more hesitant than males, which is consistent with previous findings performed in Kuwait [18,19], Jordan [20,21], Saudi Arabia [23], France [26], Ireland, and the UK [31], and the USA [34,35,36,38]. This could be because females were found to have higher trust in conspiratorial beliefs and lower trust in the COVID-19 risk [15,20]. In contrast, females in Australia were less hesitant to receive the COVID-19 vaccine [33]. Married participants were more hesitant to vaccinate, which is in agreement with that reported in Jordan [21], but in contrast to results from Saudi Arabia [22] and Greece [30], where married participants were less hesitant to vaccinate. Vaccine hesitancy was found to be more prevalent among residents in Hawalli, Al-Farwaniyah, Al-Jahra, and Mubarak Al-Kabeer, which is in disagreement with a previous study in Kuwait that reported a higher vaccine hesitancy among only Al-Jahra’s residents [19]. Participants with an intermediate monthly average income (500–1000 KD) were found to be more hesitant toward vaccination, compared with other studies, where low income was associated with vaccine hesitancy [20,23,31,32,34,36,38], despite the fact that Kuwait is offering the COVID-19 vaccine for free. Non-smoker respondents were found to be more hesitant to vaccinate which is in contrast to a previous finding reported by a study in Kuwait in which smokers were more hesitant to vaccinate [19]. Further qualitative research is required to provide a better understanding for these findings.

Respondents who were not worried about catching the COVID-19 infection were found to be more hesitant to vaccinate, which is consistent with previous results in Saudi Arabia [22], France [26], Northern Italy [28], and the USA [34,36,37,38]. Furthermore, vaccine hesitancy was found to be more prevalent among respondents who do not know if a family member has been infected or died because of COVID-19 infection. Therefore, the susceptibility and severity of the disease in the population should be addressed adequately with a clear explanation about the vaccine mechanism and the way it addresses the severity through effective educational campaigns [37]. Respondents who do not have a large extent of confidence in the Kuwait health system’s ability to handle the COVID-19 pandemic well were more hesitant to vaccinate, which is consistent with previous findings in Kuwait [18], Saudi Arabia [22], Ireland, and the UK [31]. Respondents who did not receive the influenza vaccine during the last 12 months were more hesitant to vaccinate, which is in agreement with prior findings in Kuwait [18,19], Saudi Arabia [23], Italy [27], Greece [30], the UK [32], and the USA [36,38]. Moreover, vaccine hesitancy was highest among participants who refused or elected to forego a doctor-recommended vaccine for themselves or someone they are responsible for, which is similar to previous findings in Saudi Arabia [23] and Northern Italy [28]. This could be explained as people having more doubts about vaccines in general; they are more hesitant toward the COVID-19 vaccines and other vaccines [27]. Participants who did not receive adequate information about the available COVID-19 vaccines in Kuwait by public health authorities/their healthcare providers and who do not have any first-degree family members who received or are intending to receive the vaccine were found to be more hesitant to vaccinate. These two predictors were not reported in previous similar studies because they were conducted before the vaccine had been made available. The UK study [32] reported a higher vaccine hesitancy among those with intermediate to high levels of negative attitudes towards mistrust of vaccine benefit and concerns about future unforeseen side effects, while in our study, vaccine hesitancy associated with intermediate to high levels of negative attitudes towards mistrust of vaccine benefit, concerns about commercial profiteering, preference for natural immunity, and the overall VAX score, with no association with concerns about future unforeseen side effects. This non-significant association in the present study indicates that those who were vaccinated or intending to vaccinate have concerns regarding the future unforeseen side effects that were not significantly different from those who expressed vaccine hesitancy.

The present findings highlight the need for the design of effective educational interventions to increase the receipt of the COVID-19 vaccine by the general public in Kuwait. Health authorities should design educational campaigns to focus on raising knowledge and trust in the safety and efficacy of the vaccines and the potential benefits of vaccination, and oppose the spread of false information. The clinical evidence of the safety and efficacy of the vaccines must be the key message to be delivered during these campaigns. Additionally, the predictors for vaccine hesitancy identified by the present study should be targeted in these educational interventions. The WHO recommended the use of open communication to address these concerns as one of the various approaches to increase the COVID-19 vaccine acceptability [50]. The educational interventions provided by trained community leaders who already have the general public’s trust, such as religious leaders and community groups, and the dialogue-based communication in the healthcare facilities, as well as the online platforms by the healthcare providers, have revealed promising success for decreasing vaccine hesitancy among the general population [51]. The healthcare providers, mainly physicians involved in primary and preventive care, should intensively promote COVID-19 vaccination because their recommendation to the general population has a significant impact on vaccination rates. Therefore, healthcare professionals should actively be involved in health education and communication to combat misinformation and spread evidence-based information regarding vaccine efficacy and safety transparently [52,53]. Effective communication through motivational interviewing to educate the public, correct the false information, and reinforce the vaccine benefits was reported as an evidence-based strategy to increase vaccine receipt [54]. These recommended awareness-raising approaches should be incorporated by the Kuwait health authorities in their educational interventions.

### Strengths and Limitations of the Study

The strengths of the present study include the following: (i) The large sample size, which may reduce the effect of the present bias; (ii) The calculated minimum sample size was 1120; hence, the present sample size of 4147 could provide sufficient data for the subgroups to use the multivariable logistic regression to identify the predictors for vaccine hesitancy; (iii) The conduction of the study after the vaccine has been made available, which could reflect a more accurate data for acceptability of the COVID-19 vaccine; and (iv) The evaluation of the respondents’ attitudes towards vaccines in general rather than towards a COVID-19 vaccine specifically, which may have avoided the respondents’ fears of the COVID-19 vaccine.

The interpretation of the current results should consider certain potential limitations. These include the following: (i) The convenience sampling method for data collection allowed only those who had access to social media and emails to access the survey, and the over-representation of the female gender and those who work or study in the medical field in the study sample indicate selection bias. That would have affected the external validity in terms of generalizing the present findings to the wider population in Kuwait. However, it was reported that online data collection can reach inaccessible people and offer a private setting for them to answer the survey accurately and honestly compared to face-to-face interviews [55]; (ii) The current results depend upon information given by the study participants and are open to recall bias. It is possible that respondents may over-report socially desirable behaviors or under-report socially undesirable behaviors, which could have resulted in response bias. The extent of truthful answers or verifying their claims are not possible in this type of study, where responses were taken at face value. The completion of the surveys anonymously would have minimized the over-and under-reporting. (iii) Despite that, there were no submitted surveys with not agreeing to participate in the study, non-response bias cannot be ruled out since some individuals might have simply ignored to participate after reading the invitation. It is still possible that non-participants have different views compared to participants. However, our sample size was large with very few missing data, and (iv) The data collection at one point in time (cross-sectional study design), which does not reflect any modifications over time in the participants’ acceptability of the COVID-19 vaccine.

## 5. Conclusions

Almost three-quarters (73.8%) of the participants were vaccinated or intended to receive the COVID-19 vaccine, which is higher than the previous reports from Kuwait before the vaccines were made available. Despite this high level of vaccine acceptability, 26.2% indicated their uncertainty or unwillingness to vaccinate. As the COVID-19 cases are still increasing in Kuwait, as are critical cases and deaths, the vaccination process needs to be accelerated to reach the desired herd immunity. The present findings provide a steer as to the groups that most need to be reached to increase the rates of vaccine uptake.

## Figures and Tables

**Table 1:** Sociodemographic and other characteristics of the respondents (*n* = 4147).

Characteristics	Frequency (%)
Age (years) *	
18–29	1627 (39.2)
30–49	1596 (38.5)
50-64	803 (19.4)
≥65	116 (2.8)
**Gender**	
Male	1020 (24.6)
Female	3127 (75.4)
**Marital Status**	
Single	1562 (37.7)
Married	2306 (55.6)
Divorced	213 (5.1)
Widowed	66 (1.6)
**Educational Level**	
Less than high school	43 (1.0)
High school	322 (7.8)
Diploma	557 (13.4)
University	2664 (64.2)
Postgraduate	561 (13.5)
**Employment**	
Unemployed	146 (3.5)
Retired	607 (14.6)
Housewife	109 (2.6)
Student	1064 (25.7)
Professional	782 (18.9)
Self-employed	100 (2.4)
Clerical	1339 (32.3)
**Residence area**	
Capital	1157 (27.9)
Hawalli	1243 (30.0)
Al-Farwaniyah	461 (11.1)
Al-Ahmadi	404 (9.7)
Al-Jahra	252 (6.1)
Mubarak Al-Kabeer	630 (15.2)
**Monthly average income**	
Less than 500 KD	1102 (26.6)
500–1000 KD	836 (20.1)
Greater than 1000 KD	2209 (53.3)
**Working or Studying in the Medical Field**	
Yes	1020 (24.6)
No	3127 (75.4)
**If Yes, (*n* = 1020)**	
Medical doctor	78 (7.6)
Dentist	34 (3.3)
Pharmacist	239 (23.4)
Nurse	29 (2.8)
Medical student	146 (14.3)
Dental student	32 (3.1)
Pharmacy student	207 (20.3)
Nursing Student	3 (0.3)
Other	252 (24.7)
**Self-Reported Overall Health**	
Very Good	2165 (52.2)
Good	1705 (41.1)
Fair	241 (5.8)
Poor	30 (0.7)
Very Poor	6 (0.1)
**Smoking**	
Current Smoker	562 (13.6)
Current Non-smoker	3585 (86.4)
**Having Chronic Disease(s)**	
Yes	1043 (25.2)
No	3104 (74.8)

* Percentage may not total 100% due to 5 missing responses.

**Table 2:** Information related to the COVID-19 infection (*n* = 4147).

Responses	Frequency (%)
**Feeling Worried about Catching a COVID-19 Infection**	
Yes	2461 (59.3)
No	1686 (40.7)
**Infected with COVID-19**	
Ye	739 (17.8)
No	2835 (68.4)
I do not know	573 (13.8)
**Any of their Family Members have been Infected with COVID-19**	
Yes	2752 (66.4)
No	1258 (30.3)
I do not know	137 (3.3)
**Any of Their Family Members Died because of COVID-19 Infection**	
Yes	807 (19.4)
No	3263 (78.7)
I do not know	77 (1.9)
**Sources Utilized to Obtain Information about COVID-19 Infection**	
Mass media (Television, Radio, Newspapers)	
Yes	2027 (48.9)
No	2120 (51.1)
Social media (Twitter, Instagram, Telegram, Facebook, WhatsApp)	
Yes	2850 (68.7)
No	1297 (31.3)
Scientific websites and articles	
Yes	1513 (36.5)
No	2634 (63.5)
Healthcare providers (physicians, Pharmacists, Nurses)	
Yes	1873 (45.2)
No	2274 (54.8)
Family, relatives, friends	
Yes	1085 (26.2)
No	3062 (73.8)
**Knowledge Level on COVID-19 Infection**	
Poor	94 (2.3)
Moderate	1088 (26.2)
Good	1953 (47.1)
Very Good	1012 (24.4)
**The Extent of Following Recommendations from the Health Authorities to Prevent the Spread of COVID-19?**	
Not at all	69 (1.7)
To a small extent	309 (7.5)
To a moderate extent	1561 (37.6)
To a large extent	2208 (53.2)
**The Extent of Confidence in the Kuwait Government’s Ability to Handle the COVID-19 Pandemic Well**	
Not at all	575 (13.9)
To a small extent	1050 (25.3)
To a moderate extent	1624 (39.2)
To a large extent	898 (21.6)
**The Extent of Confidence in the Kuwait Health System’s Ability to Handle the COVID-19 Pandemic Well**	
Not at all	351 (8.5)
To a small extent	883 (21.3)
To a moderate extent	1738 (41.9)
To a large extent	1175 (28.3)

**Table 3:** Reasons for uncertainty or unwillingness to vaccinate (*n* = 1086).

Reasons	Frequency (%)
I do not believe that COVID-19 is a serious infection that requires vaccination	299 (27.5)
COVID-19 infection is a conspiracy and I do not believe that it exists	122 (11.2)
I am concerned about the efficacy of the COVID-19 vaccine in preventing me from getting the infection	526 (48.4)
I am concerned about the possible side effects of the COVID-19 vaccine	821 (75.6)
I do not yet have adequate information about the vaccine to decide	355 (32.7)
The development of the COVID-19 vaccine was rushed, and it has not been thoroughly tested before approval	576 (53)
The COVID-19 vaccine has not yet been taken by many people in the public to know its side effects	370 (34.1)
Some of the physicians posted videos on social media advising against receiving the vaccine raising my suspicion	349 (32.1)
I have a strong immune system that protects me against the COVID-19 infection	178 (16.4)
I have allergies and am worried that I may have an allergic reaction to it	217 (20.0)
Others	35 (3.2)

**Table 4:** Responses to the Vaccination Attitude Examination (VAX) Scale Items (*n =* 4147).

Items	High Level of Negative Attitude *n* (%)	Intermediate Level of Negative Attitude *n* (%)	Low Level of Negative Attitude *n* (%)	Median (IQR) ^	Mean (SD) ^
I feel safe after being vaccinated	822 (19.8)	1119 (27.0)	2206 (53.2)	2 (3)	2.8 (1.7)
I can rely on vaccines to stop serious infectious diseases	623 (15.0)	1030 (24.8)	2494 (60.1)	2 (3)	2.5 (1.7)
I feel protected after getting vaccinated	757 (18.3)	1155 (27.9)	2235 (53.9)	2 (3)	2.7 (1.7)
Although most vaccines appear to be safe there may be problems that have not yet been discovered *	2032 (49.0)	1492 (36.0)	623 (15.0)	4 (3)	4.3 (1.5)
Vaccines can cause unforeseen problems in children *	1368 (33.0)	1812 (43.7)	967 (23.3)	4 (2)	3.7 (1.6)
I worry about the unknown effects of vaccines in the future *	1691 (40.8)	1298 (31.3)	1158 (27.9)	4 (4)	3.8 (1.8)
Vaccines make a lot of money for pharmaceutical companies, but do not do much for regular people *	1166 (28.1)	1539 (37.1)	1442 (34.8)	3 (3)	3.4 (1.7)
Authorities promote vaccination for financial gain, not for people’s health *	552 (13.3)	1053 (25.4)	2542 (61.3)	2 (2)	2.4 (1.6)
Vaccination programs are a big con *	421 (10.2)	840 (20.3)	2886 (69.6)	1 (2)	2.1 (1.5)
Natural immunity lasts longer than a vaccination *	1093 (26.4)	1705 (41.1)	1349 (32.5)	3 (3)	3.4 (1.7)
Natural exposure to viruses and germs gives the safest protection *	815 (19.7)	1719 (41.5)	1613 (38.9)	3 (2)	3.1 (1.6)
Being exposed to diseases naturally is safer for the immune system than being exposed through vaccination *	834 (20.1)	1529 (36.9)	1784 (43.0)	3 (3)	3 (1.7)

* Reversed score for these items to reflect the score classification of high (score 5–6), intermediate, (score 3–4), and low (score 1–2) levels of negative attitudes; ^ Responses were rated on a six-point scale from 1 “strongly agree” to 6 “strongly disagree.”

**Table 5:** Vaccination Attitude Examination (VAX) Subscales (*n* = 4147).

Subscales	High level of Negative Attitude *n* (%)	Intermediate level of Negative Attitude *n* (%)	Low level of Negative Attitude *n* (%)	Median (IQR) ^	Mean (SD) ^
Mistrust of Vaccine Benefit	734 (17.7)	1101 (26.5)	2312 (55.8)	2 (3)	2.7 (1.7)
Worries over Unforseen Future Effects	1697 (40.9)	1534 (37.0)	916 (22.1)	4 (3)	4.0 (1.7)
Concerns about Commercial Profiteering	713 (17.2)	1144 (27.6)	2290 (55.2)	2 (3)	2.6 (1.7)
Preference for Natural Immunity	914 (22.0)	1651 (39.8)	1582 (38.2)	3 (2)	3.1 (1.7)
Overall VAX score	1014 (24.5)	1358 (32.7)	1775 (42.8)	2 (3)	2.7 (1.7)

^ Responses were rated on a six-point scale from 1 “strongly agree” to 6 “strongly disagree”.

**Table 6:** Multivariable logistic regression for the predictors of uncertainty and unwillingness to vaccinate against the COVID-19 infection (*n* = 4147).

Characteristics	OR (95% CI)	*p*-Value
**Age (years)**		0.003 *
18–29	2.1 (0.8–5.6)	
30–49	3.3 (1.3–8.3)	
50–64	2.4 (1.0–5.8)	
≥65	Reference	
**Gender**		<0.001 *
Male	Reference	
Female	1.7 (1.3–2.3)	
**Marital Status**		0.014 *
Single	Reference	
Married	1.5 (1.1–2.0)	
Divorced	2.0 (1.2–3.3)	
Widowed	1.2 (0.5–3.0)	
**Employment**		0.478
Unemployed	1.3 (0.7–2.4)	
Retired	0.8 (0.5–1.2)	
Housewife	0.7 (0.4–1.2)	
Student	1.2 (0.7–1.8)	
Employed	Reference	
**Residence area**		0.001 *
Capital	Reference	
Hawalli	1.4 (1.0–1.8)	
Al-Farwaniyah	1.9 (1.3–2.6)	
Al-Ahmadi	1.1 (0.8–1.6)	
Al-Jahra	1.9 (1.3–2.9)	
Mubarak Al-Kabeer	1.7 (1.2–2.3)	
**Monthly income**		0.029 *
<500 KD	1.2 (0.8–1.8)	
500–1000 KD	1.4 (1.1–1.9)	
>1000 KD	Reference	
**Work or study in the medical field**		0.853
Ye	Reference	
No	1.0 (0.8–1.3)	
**Smoking**		0.005 *
Smoker	Reference	
Non-smoker	1.6 (1.1–2.2)	
**Have chronic disease(s)**		0.239
Yes	Reference	
No	0.9 (0.7–1.1)	
**Feeling worried about catching a COVID-19 infection**		<0.001 *
Yes	Reference	
No	1.7 (1.4–2.1)	
**Infected with COVID-19**		0.095
Yes	Reference	
No	0.9 (0.7–1.2)	
I do not know	1.2 (0.9–1.8)	
**Any of their family members have been infected with COVID-19**		0.014 *
Yes	Reference	
No	1.0 (0.8–1.3)	
I do not know	2.1 (1.3–3.6)	
**Any of their family members died because of COVID-19 infection**		0.019*
Yes	Reference	
No	1.1 (0.9–1.4)	
I do not know	2.4 (1.2–4.9)	
**Obtaining knowledge about COVID-19 infection from mass media**		0.830
Yes	Reference	
No	1.0 (0.8–1.3)	
**Obtaining knowledge about COVID-19 infection from social media**		0.591
Yes	Reference	
No	1.1 (0.9–1.3)	
**Obtaining knowledge about COVID-19 infection from healthcare providers**		0.070
Yes	Reference	
No	1.2 (0.9–1.5)	
**Obtaining knowledge about COVID-19 infection from family, relatives, and friends**		0.278
Yes	Reference	
No	0.9 (0.7–1.1)	
**knowledge level on COVID-19 infection**		0.712
Poor	Reference	
Moderate	1.4 (0.7–2.6)	
Good	1.4 (0.7–2.6)	
Very Good	1.5 (0.8–2.9)	
**The extent of following recommendations from the health authorities to prevent the spread of COVID-19**		0.413
Not at all	1.3 (0.6–3.1)	
To a small extent	1.1 (0.8–1.6)	
To a moderate extent	0.9 (0.7–1.1)	
To a large extent	Reference	
**The extent of confidence in the Kuwait Government’s ability to handle the COVID-19 pandemic well**		0.062
Not at all	1.6 (0.9–2.6)	
To a small extent	1.3 (0.9–2.0)	
To a moderate extent	1.0 (0.7–1.4)	
To a large extent	Reference	
**The extent of confidence in the Kuwait health system’s ability to handle the COVID-19 pandemic well**		0.002 *
Not at all	2.3 (1.6–3.4)	
To a small extent	2.1 (1.4–3.2)	
To a moderate extent	1.6 (1.1–2.2)	
To a large extent	Reference	
**Received influenza (flu) vaccine during the last 12 months**		<0.001 *
Yes	Reference	
No	8.3 (6.1–11.5)	
**Refused or elected to forego a doctor-recommended vaccine for them or someone they are responsible for**		<0.001 *
Yes	Reference	
No	0.4 (0.3–0.6)	
**Aware that there are multiple vaccines available for COVID-19**		0.262
Yes	Reference	
No	1.3 (0.8–2.2)	
**Received adequate information from the public health authorities/their healthcare providers about the COVID-19 vaccines available in Kuwait**		0.015 *
Yes	Reference	
No	1.3 (1.1–1.6)	
**Any of their first-degree family members received or are intending to receive the COVID-19 vaccine**		<0.001 *
Yes	Reference	
No	4.3 (3.1–5.9)	
**Mistrust of vaccine benefit**		<0.001 *
High level of negative attitude	12.9 (9.8–16.9)	
Intermediate level of negative attitude	4.2 (3.3–5.4)	
Low level of negative attitude	Reference	
**Worries over unforeseen future effects**		0.372
High level of negative attitude	1.1 (0.8–1.5)	
Intermediate level of negative attitude	0.9 (0.6–1.3)	
Low level of Negative Attitude	Reference	
**Concerns about commercial profiteering**		<0.001 *
High level of negative attitude	2.3 (1.8–3.1)	
Intermediate level of negative attitude	1.3 (1.0–1.6)	
Low level of negative attitude	Reference	
**Preference for natural immunity**		<0.001 *
High level of negative attitude	2.0 (1.5–2.6)	
Intermediate level of negative attitude	1.6 (1.2–2.1)	
Low level of negative attitude	Reference	
**Overall VAX Attitude**		<0.001 *
High level of negative attitude	12.9 (9.8–17.0)	
Intermediate level of negative attitude	4.1 (3.3–5.6)	
Low level of negative attitude	Reference	

* p-value of < 0.05 significant predictor

## Data Availability

Available from the corresponding author upon request.

## References

[1] World Health Organization Naming the Coronavirus Disease (COVID-19) and the Virus that Causes It. https://www.who.int/emergencies/diseases/novel-coronavirus-2019/technical-guidance/naming-the-coronavirus-disease-(covid-2019)-and-the-virus-that-causes-it.

[2] Ling Z., Xu X., Gan Q., Zhang L., Luo L., Tang X., Liu J. (2020). Asymptomatic SARS-CoV-2 infected patients with persistent negative CT findings. Eur. J. Radiol..

[3] World Health Organization Coronavirus Disease 2019 (COVID-19) Situation Report-51. https://www.who.int/docs/default-source/coronaviruse/situation-reports/20200311-sitrep-51-covid-19.pdf?sfvrsn=1ba62e57_10.

[4] Worldometer COVID-19 Coronavirus Pandemic. https://www.worldometers.info/coronavirus/?utm_campaign=homeAdvegas1?%20.

[5] DeRoo S.S., Pudalov N.J., Fu L.Y. (2020). Planning for a COVID-19 Vaccination Program. JAMA.

[6] World Health Organization Vaccines: Coronavirus Disease (COVID-19). https://extranet.who.int/pqweb/vaccines/covid-19-vaccines.

[7] World Health Organization WHO Coronavirus (COVID-19) Dashboard. https://covid19.who.int/?gclid=Cj0KCQjwl_SHBhCQARIsAFIFRVW6d2OmANY0ZinWfzlhFXYEr7wXapu9T4VIGZPdoyXAmBhUVbxbbuEaAop-EALw_wcB.

[8] Times Kuwait Rapid Vaccination Rate May Lead to Herd Immunity Soon. https://www.timeskuwait.com/news/rapid-vaccination-rate-may-lead-to-herd-immunity-soon/.

[9] World Health Organization Global Immunization Data. https://www.who.int/immunization/monitoring_surveillance/global_immunization_data.pdf?ua=1.

[10] Larson H.J., Jarrett C., Eckersberger E., Smith D.M., Paterson P. (2014). Understanding vaccine hesitancy around vaccines and vaccination from a global perspective: A systematic review of published literature, 2007–2012. Vaccine.

[11] Bianco A., Mascaro V., Zucco R., Pavia M. (2019). Parent perspectives on childhood vaccination: How to deal with vaccine hesitancy and refusal?. Vaccine.

[12] Napolitano F., D’Alessandro A., Angelillo I.F. (2018). Investigating Italian parents’ vaccine hesitancy: A cross-sectional survey. Hum. Vaccines Immunother..

[13] Dubé È., Farrands A., Lemaitre T., Boulianne N., Sauvageau C., Boucher F.D., Tapiero B., Quach C., Ouakki M., Gosselin V. (2019). Overview of knowledge, attitudes, beliefs, vaccine hesitancy and vaccine acceptance among mothers of infants in Quebec, Canada. Hum. Vaccine Immunother..

[14] World Health Organization.Ten Threats to Global Health in 2019. https://www.who.int/news-room/spotlight/ten-threats-to-global-health-in-2019.

[15] Sallam M. (2021). COVID-19 Vaccine Hesitancy Worldwide: A Concise Systematic Review of Vaccine Acceptance Rates. Vaccines.

[16] Robinson E., Jones A., Lesser I., Daly M. (2021). International estimates of intended uptake and refusal of COVID-19 vaccines: A rapid systematic review and meta-analysis of large nationally representative samples. Vaccine.

[17] Lazarus J.V., Ratzan S.C., Palayew A., Gostin L.O., Larson H.J., Rabin K., Kimball S., El-Mohandes A. (2021). A global survey of potential acceptance of a COVID-19 vaccine. Nat. Med..

[18] AlAwadhi E., Zein D., Mallallah F., Bin Haider N., Hossain A. (2021). Monitoring COVID-19 Vaccine Acceptance in Kuwait during the Pandemic: Results from a National Serial Study. Risk Manag. Healthc. Policy.

[19] Alqudeimat Y., Alenezi D., AlHajri B., Alfouzan H., Almokhaizeem Z., Altamimi S., Almansouri W., Alzalzalah S., Ziyab A.H. (2021). Acceptance of a COVID-19 Vaccine and Its Related Determinants among the General Adult Population in Kuwait. Med. Princ. Pract..

[20] Sallam M., Dababseh D., Eid H., Al-Mahzoum K., Al-Haidar A., Taim D., Yaseen A., Ababneh N.A., Bakri F.G., Mahafzah A. (2021). High Rates of COVID-19 Vaccine Hesitancy and Its Association with Conspiracy Beliefs: A Study in Jordan and Kuwait among Other Arab Countries. Vaccines.

[21] Al-Qerem W.A., Jarab A.S. (2021). COVID-19 Vaccination Acceptance and Its Associated Factors Among a Middle Eastern Population. Front. Public Health.

[22] Al-Mohaithef M., Padhi B.K. (2020). Determinants of COVID-19 Vaccine Acceptance in Saudi Arabia: A Web-Based National Survey. J. Multidiscip. Healthc..

[23] Alfageeh E.I., Alshareef N., Angawi K., Alhazmi F., Chirwa G.C. (2021). Acceptability of a COVID-19 Vaccine among the Saudi Population. Vaccines.

[24] Lin Y., Hu Z., Zhao Q., Alias H., Danaee M., Wong L.P. (2020). Understanding COVID-19 vaccine demand and hesitancy: A nationwide online survey in China. PLoS Negl. Trop. Dis..

[25] Harapan H., Wagner A.L., Yufika A., Winardi W., Anwar S., Gan A.K., Setiawan A.M., Rajamoorthy Y., Sofyan H., Mudatsir M. (2020). Acceptance of a COVID-19 Vaccine in Southeast Asia: A Cross-Sectional Study in Indonesia. Front. Public Health.

[26] Schwarzinger M., Watson V., Arwidson P., Alla F., Luchini S. (2021). COVID-19 vaccine hesitancy in a representative working-age population in France: A survey experiment based on vaccine characteristics. Lancet Public Health.

[27] Caserotti M., Girardi P., Rubaltelli E., Tasso A., Lotto L., Gavaruzzi T. (2021). Associations of COVID-19 risk perception with vaccine hesitancy over time for Italian residents. Soc. Sci. Med..

[28] Reno C., Maietti E., Fantini M.P., Savoia E., Manzoli L., Montalti M., Gori D. (2021). Enhancing COVID-19 Vaccines Acceptance: Results from a Survey on Vaccine Hesitancy in Northern Italy. Vaccines.

[29] Bendau A., Plag J., Petzold M.B., Ströhle A. (2021). COVID-19 vaccine hesitancy and related fears and anxiety. Int. Immunopharmacol..

[30] Kourlaba G., Kourkouni E., Maistreli S., Tsopela C.G., Molocha N.M., Triantafyllou C., Koniordou M., Kopsidas I., Chorianopoulou E., Maroudi-Manta S. (2021). Willingness of Greek general population to get a COVID-19 vaccine. Glob. Health Res. Policy.

[31] Murphy J., Vallières F., Bentall R.P., Shevlin M., McBride O., Hartman T.K., McKay R., Bennett K., Mason L., Gibson-Miller J. (2021). Psychological characteristics associated with COVID-19 vaccine hesitancy and resistance in Ireland and the United Kingdom. Nat. Commun..

[32] Paul E., Steptoe A., Fancourt D. (2021). Attitudes towards vaccines and intention to vaccinate against COVID-19: Implications for public health communications. Lancet Reg. Health-Eur..

[33] Seale H., Heywood A.E., Leask J., Sheel M., Durrheim D.N., Bolsewicz K., Kaur R. (2021). Examining Australian public perceptions and behaviors towards a future COVID-19 vaccine. BMC Infect. Dis..

[34] Khubchandani J., Sharma S., Price J.H., Wiblishauser M.J., Sharma M., Webb F.J. (2021). COVID-19 Vaccination Hesitancy in the United States: A Rapid National Assessment. J. Community Health.

[35] Malik A.A., McFadden S.M., Elharake J., Omer S.B. (2020). Determinants of COVID-19 vaccine acceptance in the US. EClinicalMedicine.

[36] Coe A.B., Elliott M.H., Gatewood S.B.S., Goode J.R., Moczygemba L.R. (2021). Perceptions and predictors of intention to receive the COVID-19 vaccine. Res. Soc. Adm. Pharm..

[37] Viswanath K., Bekalu M., Dhawan D., Pinnamaneni R., Lang J., McLoud R. (2021). Individual and social determinants of COVID-19 vaccine uptake. BMC Public Health.

[38] Kelly B.J., Southwell B.G., McCormack L.A., Bann C.M., MacDonald P.D.M., Frasier A.M., Bevc C.A., Brewer N.T., Squiers L.B. (2021). Predictors of willingness to get a COVID-19 vaccine in the U.S. BMC Infect. Dis..

[39] Reiter P.L., Pennell M.L., Katz M.L. (2020). Acceptability of a COVID-19 vaccine among adults in the United States: How many people would get vaccinated?. Vaccine.

[40] Soares P., Rocha J.V., Moniz M., Gama A., Laires P.A., Pedro A.R., Dias S., Leite A., Nunes C. (2021). Factors Associated with COVID-19 Vaccine Hesitancy. Vaccines.

[41] Daly M., Jones A., Robinson E. (2021). Public Trust and Willingness to Vaccinate Against COVID-19 in the US From October 14, 2020, to March 29, 2021. JAMA.

[42] World Population Review State of Kuwait: General Statistics Bureau World Population Prospects (2021 Revision). United Nations Population Estimates and Projections. https://worldpopulationreview.com/countries/kuwait-population.

[43] Covidvax Live COVID-19 Vaccination Tracker-Kuwait. https://covidvax.live/location/kwt.

[44] Dupont W., Plummer W.J. Power and Sample Size Calculation. https://biostat.app.vumc.org/wiki/Main/PowerSampleSize.

[45] Martin L.R., Petrie K.J. (2017). Understanding the Dimensions of Anti-Vaccination Attitudes: The Vaccination Attitudes Examination (VAX) Scale. Ann. Behav. Med..

[46] Wood L., Smith M., Miller C.B., O’Carroll R.E. (2019). The Internal Consistency and Validity of the Vaccination Attitudes Examination Scale: A Replication Study. Ann. Behav. Med..

[47] Bendel R.B., Afifi A.A. (1977). Comparison of Stopping Rules in Forward “Stepwise” Regression. J. Am. Stat. Assoc..

[48] Mickey R.M., Greenland S. (1989). The impact of confounder selection criteria on effect estimation. Am. J. Epidemiol..

[49] Randolph H.E., Barreiro L.B. (2020). Herd Immunity: Understanding COVID-19. Immunity.

[50] World Health Organization Behavioural Considerations for Acceptance and Uptake of COVID-19 Vaccines. https://www.who.int/news/item/21-12-2020-behavioural-considerations-for-acceptance-and-uptake-of-covid-19-vaccines.

[51] European Centre for Disease Prevention and Control (ECDC) Catalogue of Interventions Addressing Vaccine Hesitancy. https://www.ecdc.europa.eu/sites/portal/files/documents/Catalogue-interventions-vaccine-hesitancy.pdf.

[52] Di Giuseppe G., Pelullo C.P., Della Polla G., Pavia M., Angelillo I.F. (2021). Exploring the Willingness to Accept SARS-CoV-2 Vaccine in a University Population in Southern Italy, September to November 2020. Vaccines.

[53] Wang J., Lu X., Lai X., Lyu Y., Zhang H., Fenghuang Y., Jing R., Li L., Yu W., Fang H. (2021). The Changing Acceptance of COVID-19 Vaccination in Different Epidemic Phases in China: A Longitudinal Study. Vaccines.

[54] MacDonald N.E., Butler R., Dubé E. (2018). Addressing barriers to vaccine acceptance: An overview. Hum. Vaccine Immunother..

[55] Cantrell M.A., Lupinacci P. (2007). Methodological issues in online data collection. J. Adv. Nurs..

